# Next generation therapeutics for Alzheimer's disease

**DOI:** 10.1002/emmm.201202307

**Published:** 2013-05-23

**Authors:** Dale E Bredesen, Varghese John

**Affiliations:** 1 Buck Institute for Research on AgingNovato, CA, USA; 2 Department of Neurology, University of CaliforniaSan Francisco, CA, USA

**Keywords:** aging, Alzheimer's disease, chronic illnesses, dementia, multitherapy

## Abstract

To date, no truly effective therapy has been developed for Alzheimer's disease or mild cognitive impairment. In searching for new approaches that may succeed where previous ones have failed, it may be instructive to consider the successful therapeutic developments for other chronic illnesses such as cancer and human immunodeficiency virus.

## The current status

Dementia is one of the most significant global healthcare problems, with over 30 million symptomatic individuals, and many more likely to be in the decades-long pre-symptomatic phases (World Alzheimer Report, 2009, http://www.alz.co.uk/research/files/WorldAlzheimerReport.pdf). In the United States alone, over five million people suffer from Alzheimer's disease (AD), at an estimated annual cost of $200 billion, and a projection for 13 million patients by 2050. The high prevalence of AD is of particular concern because of the lack of success in developing effective therapeutics: in comparison to most classes of disease – from neoplasia to cardiovascular and cerebrovascular disease to osteoporosis to diabetes to mental illness – therapeutic development for AD has been, to date, a failure. Why?

The answer to this critical question is likely to be multi-faceted, and at least two of the more obvious facets relate to the similarities between AD and other chronic illnesses. First, there may be lessons to be learned from the successful development of therapeutics for other chronic illnesses, such as AIDS (acquired immunodeficiency syndrome), cancer, multiple sclerosis, type II diabetes mellitus and cardiovascular disease. HIV (human immunodeficiency virus) infection was transformed from a minimally treatable disease – similar to the current state of AD treatments – to a clearly treatable and chronically manageable disease with the introduction of combination therapy (HAART, highly active anti-retroviral therapy), in preference to monotherapy. Similarly, a major advance in oncology occurred with the introduction of combination chemotherapy (Frei et al, 1965), which has become the standard of care for numerous types of cancer. It is therefore noteworthy that, of the over 40 ongoing Phase 1, Phase 2 and Phase 3 clinical trials for Alzheimer's disease, virtually all involve monotherapeutic approaches (Mangialasche et al, 2010; Piau et al, 2011; Potter, 2010; Kushwah et al, 2012). Given the historical precedents, perhaps such an approach will not turn out to be the optimal one for the treatment of AD.

» Is it possible that a comparison of the common features of the most frequent age-associated chronic illnesses may help provide insight into AD pathogenesis, and suggest novel therapeutic directions? «

## Feasibility of approvals

However, if the optimal therapeutic approach to AD does indeed turn out to involve a multi-component cocktail, an obvious consideration relates to the development and approval processes required for a cocktail approach: in the case of HIV treatment, each of the cocktail's constituents exerts a significant, albeit modest, effect on HIV infection. However, considering the numerous mechanisms identified as underlying AD pathogenesis, it is conceivable that many more than three different therapeutic agents will be required for optimal treatment of AD. Of even greater concern is the possibility that none of the components of the optimal therapeutic cocktail will turn out to exert a significant therapeutic effect when administered alone. How, then, would the optimal combination be identified, and ultimately approved for clinical use? Significant modernization of the current translational approach, clinical trial methodology and approval process may be required to render the optimization and approval of such a therapeutic cocktail feasible.

## Emerging pathogenesis: effect on therapeutic development

A second lesson to be learned involves the potential relationship between the pathogenesis of AD and the pathogenetic processes underlying other chronic disease states such as osteoporosis and neoplasia. Is it possible that the therapeutic failure to date in AD may have resulted, at least in part, from an incomplete understanding of the etiology and pathogenesis of AD? Any accurate theory of AD must explain a number of features (Table 1): for example, why is AD risk increased by such disparate factors as the ApoE ε4 allele, early oophorectomy (ovarian removal, for example as part of a total hysterectomy), metabolic syndrome, head trauma, inflammatory processes and hyperhomocysteinemia? What is the physiological role(s) of Aβ peptides, and how does it relate to the pathophysiology of AD? Moreover, recent results from a number of sources must be taken into account by any new theory: for example, both Aβ and tau may function as prions (de Calignon et al, 2012; Eisele et al, 2009; Yang et al, 1995). The four peptides derived from the amyloidogenic processing of β-amyloid precursor protein (APP) – sAPPβ, Aβ, Jcasp and C31 – have been shown to mediate neurite retraction, synaptic inhibition, caspase activation and programmed cell death (Bertrand et al, 2001; Lu et al, 2000, 2003; Nikolaev et al, 2009); whereas the two peptides derived from the non-amyloidogenic processing of APP – sAPPα and αCTF – support neurite extension, inhibit Aβ production, inhibit caspase activation and inhibit programmed cell death (Deyts et al, 2012; Guo et al, 1998; Tian et al, 2010). Thus, APP appears to function as a molecular switch, mediating plasticity-related processes and AD is associated, whether causally or incidentally, with an increase in the ratio of the neurite-retractive peptides to the neurite-extending peptides. Reducing this ratio, whether by affecting BACE (β-site APP cleaving enzyme) or other cleavage of APP, appears to mitigate the AD severity (Bredesen et al, 2010; Galvan et al, 2006; Jonsson et al, 2012).

**Table 1:** Features of Alzheimer's disease to be explained by any accurate theory

Lack of successful therapeutic development to date.
The remarkable diversity of risk factors for AD.
The high prevalence of AD in the elderly.
The mechanism(s) by which ApoE4 increases risk for AD.
The physiological role(s) of Aβ peptides.
The anatomic pattern of spread of AD pathology.
The association of plastic brain regions with AD pathology.
Why some people, and transgenic mice, collect large amounts of Aβ peptide without displaying symptoms of AD.
The relationship between Aβ and tau pathology.
The α7 paradox (*i.e.* α7 has been reported to mediate both enhancement and inhibition of neurodegeneration) (Dziewczapolski et al, 2009; O'Neill et al, 2002)

» AD, like other chronic illnesses, is an age-associated network imbalance that features many underlying mechanisms, and many or all of these mechanisms may need to be addressed therapeutically for optimal clinical efficacy. «

## Alzheimer's disease, osteoporosis and cancer

Is it possible that a comparison of the common features of the most frequent age-associated chronic illnesses may help provide insight into AD pathogenesis, and suggest novel therapeutic directions? For both osteoporosis and neoplasia, there is a fundamental, age-associated imbalance between dynamically opposed physiological processes: in the case of osteoporosis, the imbalance is between osteoblastic and osteoclastic activity, physiological mediators of bone development, remodelling and repair; whereas, in the case of neoplasia, the imbalance is between oncogene and tumour suppressor gene activity, physiological mediators of tissue development, remodelling and repair. In the case of neoplasia, there is an added feature of positive feedback, in that a rare somatic mutation may be selected in a Darwinian fashion by the cellular survival advantage that it confers. By analogy, in Alzheimer's disease, there is a fundamental, age-associated imbalance between the dynamically opposed physiological processes that mediate plasticity, *i.e.* between “synaptoblastic” and “synaptoclastic” activity, physiological mediators of synaptic development, maintenance, repair and remodelling, signaled via APP, its derivative peptides, ApoE and tau and modulated by all of the many disparate factors associated with Alzheimer's disease. Furthermore, just as for neoplasia, positive feedback selects and amplifies the disease process; however, whereas in oncogenesis, the positive feedback occurs at the cellular level, in Alzheimer's disease, the positive feedback occurs at the molecular species level, in the form of prionic loops.

What would be the therapeutic implications of such an analogy between Alzheimer's disease and these other common chronic, age-associated illnesses? One implication would be that the treatment of AD might be enhanced by taking into account the following general principles:

- AD, like other chronic illnesses, is an age-associated network imbalance that features many underlying mechanisms, and many or all of these mechanisms may need to be addressed therapeutically for optimal clinical efficacy. For example, the association of Alzheimer's disease with low vitamin D intake (Annweiler et al, 2013), coupled with the neuroprotective effects of vitamin D, suggest that optimizing vitamin D serum concentration may be required for optimal therapeutic response. Similarly, combining BACE inhibition with a tau phosphorylation inhibitor may turn out to be preferable to the use of either alone.
- Just as for other chronic illnesses such as cardiovascular disease, upstream targets are preferable to downstream targets, although both may need to be combined for optimal results. For example, if the precipitant of imbalanced APP processing is a reduction in estrogen binding to its receptors, then treatment that fails to include estrogen may be sub-optimal.
- Just as for other chronic illnesses such as cardiovascular disease, prevention and pre-symptomatic treatment are preferable to treatment later in the pathogenetic process. Indeed, since AD is a multi-prionic disease, more extensive combinations of therapeutics may be required late in the disease process than early. For example, prevention may not require a tau phosphorylation inhibitor, whereas optimal treatment of AD may require such an inhibitor.
- Rather than focusing on monotherapeutics, the optimal approaches may involve systems of therapeutics, which include both pharmacological and non-pharmacological components. For example, if synaptic reconstruction and maintenance form parts of the optimal treatment for AD, and inflammation is to be minimized, autophagy activated (periodically, perhaps), neurotrophic factors normalized, stress minimized, Aβ oligomerization inhibited, Aβ clearance increased, ApoE4-mediated signals reduced, tau phosphorylation reduced, prionic tau amplification blocked, memory loss reversed, cholinergic neurotransmission restored and overall network balance restored; then multiple factors may require normalization, enhancement, or administration, such as hormonal balance, vitamin D3, C-reactive protein (and other inflammation-related markers), homocysteine, sleep and melatonin, citicoline (citidine-5-diphosphocholine), specific antioxidants, diet (including specific periods of fasting, avoidance of high glycemic index foods and saturated fats, etc.), exercise, stress, omega-3 fatty acids and resolvins (Mizwicki et al, 2013) and other network components. Most of the factors of which such a system is comprised have already been shown to exert modest effects (trends that often have not reached statistical significance) on AD or animal models of AD, but there has been little evaluation of such a complete system. However, one of the interesting potential outcomes of including such a therapeutic system approach is that it may allow drug candidates that failed in monotherapeutic clinical trials to demonstrate beneficial effects when used as part of the system.

Rather than focusing on monotherapeutics, the optimal approaches may involve *systems* of therapeutics, which include both pharmacological and non-pharmacological components. «

Thus, the optimal prevention and treatment of AD and MCI (mild cognitive impairment) may ultimately be informed by the precedents set during development of successful therapeutics for other chronic illnesses such as cardiovascular disease, osteoporosis and cancer. Although the development and optimization of systems of therapeutics would require radical modernization and streamlining of the current complex structure involved with drug development, approval and administration, the increasing gravity of the failure to develop effective therapeutics for Alzheimer's disease argues that such therapeutic systems should be considered thoughtfully.
